# Multifunctional all-in-one adhesive hydrogel for the treatment of perianal infectious wounds

**DOI:** 10.3389/fbioe.2022.989180

**Published:** 2022-09-30

**Authors:** Ge Yin, Jingyue Wang, Xiao Wang, Yu Zhan, Xuegui Tang, Qie Wu, Xian Wang, Lijuan Du, Xiong Lu

**Affiliations:** ^1^ Department of Anorectal, Affiliated Hospital of Southwest Jiaotong University, Chengdu, China; ^2^ Department of Anorectal, Chengdu Thrid People’s Hospital, Chengdu, China; ^3^ School of Materials Science and Engineering, Southwest Jiaotong University, Chengdu, China; ^4^ Department of Anorectal, Affiliated of Integrative Chinese and Western Medicine of Chengdu University of Traditional Chinese Medicine, Chengdu, China; ^5^ Department of Anorectal, Chengdu First People’s Hospital, Chengdu, China; ^6^ Department of Anorectal, Affiliated Hospital of North Sichuan Medical College, Nanchong, China

**Keywords:** multifunctional adhesive hydrogel, antibacterial hydrogel dressing, hemostasis, infected wound healing, traditional Chinese medicine nanoparticles

## Abstract

Postoperative wound of perianal infectious disease represents a common but unique refractory wound in clinical practice. The reasons that hinder the wound healing process include not only the severe bacterial infection of the wound itself and the narrow and deep shape of the wound, but also its frequent bacterial contact. Therefore, the development of biofunctional dressings to aid in therapy is essential. In this study, we synthesized a new type of dressing comprising a hydrogel host based on the Schiff base principle and catechol groups between polydopamine, oxidized dextran, and quaternized chitosan, and then loaded it with traditional Chinese medicine molecules. These formed an integrated hydrogel for accelerated wound repair in a perianal infection model. The prepared hydrogels exhibited excellent wet tissue adhesion, antifouling, morphological variability, suitable swelling properties, and complete degradability, as well as remarkable contact antibacterial ability and the ability to rapidly scavenge free radicals. Hemostatic experiments showed excellent hemostatic properties, as the integrated hydrogel could instantly gel to seal the hemorrhage. Hemocompatibility and *in vitro* cell experiments showed that the integrated hydrogel had good biosafety and significantly promoted cell proliferation, which in turn accelerated the repair of infected whole cortexes in rats. A histomorphological evaluation showed that the integrated hydrogel promoted the recovery of normal anatomical tissue in rats by promoting the formation of collagen fibers and inhibiting inflammation. The results showed that this multifunctional integrated hydrogel has great potential for the treatment of continuously infected skin regeneration, providing a promising therapeutic strategy for postoperative wound healing in perianal infectious diseases.

## 1 Introduction

Perianal infectious disease is a common anorectal disease, mainly caused by local purulent infection of the anal sinus, anal gland, perianal space tissue, and soft tissue in the sacrococcygeal region (Mascagni et al., 2019). The latest domestic and international statistics show a high incidence of perianal infectious diseases (Hsieh et al., 2019). The annual incidence of perianal abscesses is estimated to be 17–20 cases per 100,000 people (Adamo et al., 2016), and may actually be higher. Once the clinical diagnosis is confirmed, immediate surgical treatment is recommended to prevent the disease from aggravating (Han et al., 2009). The current surgical method is mainly debridement, and the postoperative wound is an open, complex, and large incision (Malik and Nelson, 2008). In contrast to other superficial infection wounds, perianal wounds are often deep and frequently exposed to pollutants, owing to the special anatomic characteristics and location of the anus (Garg and Garg, 2015). Frequent daily cleaning can make wounds difficult to heal and prone to bleeding (Buchmann, 1990), infection (Pearce et al., 2016), pain (Seow et al., 1991), delayed healing, poor healing (Gokce and Gokce, 2020), and other complications. Medical dressings are typically used to accelerate the healing.

Currently, the most commonly used dressings in clinics are gauze dressings (Handfield-Jones et al., 1988), as they are widely available and inexpensive. However, such dressings not only cannot effectively block the invasion of external pathogens, but also take too long to stop bleeding, and lack adhesion. Moreover, dry dressings can easily destroy the growth factors that promote healing, and dressing changes can easily cause secondary damage, making the effect of adjuvant therapy unsatisfactory. In an in-depth study of the pathophysiology of the wound healing process, Zhang and J. Wu (Zhang et al., 2021) modified the surface of a gauze and used paraffin-coated hydrophobic modified hemostatic zeolite gauze to enhance its hemostasis. However, this effect also weakened the adhesion of the gauze to the tissue. Alginate dressings (Hu and Lin, 2022), represented by new wet dressings, are an updated strategy for wound healing dressings. However, these dressings only solve a certain aspect of the problem. Their application is still limited to their single function. In addition, they show incomplete degradation, fixed shapes, poor adhesion, and ease in falling off (Hu and Lin, 2022), making them unable to fully meet the needs of the complex and changeable wound applications around the anus. Therefore, there is an urgent need for the development of fully degradable, morphologically variable, highly adhesive, antibacterial, and healing-promoting medical dressings.

Owing to the three-dimensional aggregated network-like structure of the extracellular matrix, hydrogels (Xiong et al., 2021; Crunkhorn, 2020) have received increasing attention in biomedical applications in recent years, especially for tissue repair and regeneration. These mainly use external conditions such as ultraviolet (UV) light (Lim et al., 2021), co-initiators (Jiang et al., 2022), and enzyme-catalyzed reactions (Liang et al., 2019) to form stable covalent bonds between polymers and build hydrogel networks. Among these preparation strategies, the formation of hydrogel networks based on the dynamic Schiff base principle is very attractive (Du et al., 2019), owing to its rapid gelation (Balakrishnan et al., 2017) and self-healing (Giano et al., 2014) without external stimulation under physiological conditions. It has good fatigue resistance and biocompatibility, and can accelerate degradation in the weakly acidic environment of bacterial infections (Li et al., 2021); therefore, it has been used in biomedical applications such as drug delivery systems, wound healing, tissue regeneration, and tissue adhesion agents (Su et al., 2021; Li et al., 2014; Li et al., 2020).

In addition to physical properties, the direct chemical stimuli from biological contact also significantly influences the therapeutic effects (Yin et al., 2021). Natural polymers are widely used in tissue repair and regeneration because of their excellent biocompatibility, low immunogenicity, and biodegradability (Tengling et al., 2021; Pollot et al., 2017; Gul et al., 2021). Both chitosan (CS) and dextran are polysaccharides widely present in the natural environment, and CS (Tian et al., 2020) is widely used owing to its excellent hemostatic properties, tissue adhesion properties, and inherent antibacterial activity. However, the limited solubility of CS in water and other solvents limits its application in many fields. Cheah et al. (Cheah et al., 2018) used quaternized CS (QCS) to prepare nanofiber membranes, which not only improved the solubility of the CS, but also greatly enhanced its inherent antibacterial activity, providing the CS with additional application opportunities. Dextran has a large number of pendant hydroxyl groups and can be easily modified. Dextran-based hydrogels have been shown to significantly promote cardiovascular formation and skin regeneration *in vivo* (Qiu et al., 2021; Guan et al., 2021). Recently, polyphenols structurally similar to the adhesion proteins secreted by marine mussels have been shown to have strong adhesion to various substrates (Lee et al., 2007), especially by enhancing the cell adhesion and proliferation (Han et al., 2015). Owing to this great advantage, polyphenols are often used as modification materials to improve hydrogel performance. In the hydrogel system studied by Tang (Tang et al., 2021), polyphenols were used to modify CS, and significantly improved the adhesion properties of the hydrogel. Therefore, mussel-inspired materials have broad application prospects in the field of biomedicine, particularly for enhancing tissue adhesion.

Rapid wound healing and dysplasia are the main concerns for persistently infected full-thickness wounds, e.g., those represented by the postoperative wounds of perianal infectious diseases (Sahnan et al., 2017; Di Falco et al., 1986; Campos et al., 2021; Jia-Zi et al., 2016). Dragon’s blood (Nakashima et al., 2009) is a precious traditional Chinese medicine (TCM) variety containing many chemical components, mainly esters, flavonoids, saponins, and phenols. Dragon’s blood is a flavonoid extracted from draconis. Because it is very unstable when it exists in the form of a monomer, it easily decomposes; therefore, it often exists in the form of a specific salt called dracolin perchloric acid salt (DP). Recent studies have confirmed that DP can promote fibroblast proliferation, collagen formation, and inhibit scar growth; accordingly, it is widely used in scarless wound healing (Jiang et al., 2018; Jiang et al., 2017; Lu et al., 2021; Xiong et al., 2022). Moreover, it is often used as adjuvant treatment for refractory wounds (Lingli et al., 2020). Foroogh Namjoyan (Namjoyan et al., 2016) et al. made Dragon’s blood into a paste for clinical use. Although it produced evident adjuvant therapy effects, the frequent dressing changes not only wasted precious Chinese herbal medicines, but also caused a series of discomforts to the patients. A scientific drug-release system can solve this problem.

In this study, the main body of a hydrogel was constructed by the Schiff base reaction and catechol groups between polydopamine (PDA), oxidized dextran (OD), and QCS, and then was loaded with DP-containing nanoparticles (NPs) designed to form a comprehensive hydrogel. QCS gives hydrogels better inherent antibacterial properties compared with CS due to its enhanced antibacterial properties and easy solubility. PDA introduces polyphenols into the three-dimensional network to give polysaccharide based hydrogels better tissue adhesion and free radical scavenging properties. More importantly, for the first time, we loaded DP, a traditional Chinese medicine molecule, into nanoparticles and slowly released it, thus cooperating with hydrogels to promote scar free regeneration of skin and soft tissues, In this thesis, a natural degradable multifunctional self-adhesive hydrogel dressing inspired by plant was designed. The preparation conditions were mild, the method was simple, and the matrix materials were widely used. As a wound dressing with multiple biological activities, the hydrogel will have potential in the application of persistently infected full-thickness defect animal model . The rheological properties, adhesion, swelling, degradation rate, drug release, and other experiments of comprehensive hydrogels to understand the physical properties of hydrogels; through blood compatibility and cytotoxicity experiments to prove the biological properties of hydrogels Safety, and further explore the antibacterial and hemostatic properties of the hydrogel through *in vitro* antibacterial experiments and liver hemostatic models. Finally, we explored the biological properties of the synergistic effect between the hydrogel and nanoparticle-encapsulated TCM molecules for promoting efficient wound healing in a persistently infected full-thickness defect animal model.

## 2 Materials and methods

### 2.1 Materials

Dopamine hydrochloride (DA-HCl), Dextran, chitosan (CS) and bovine serum albumin (BSA)were purchased from Sigma-Aldrich (St. Louis, MO, United States). Ethanol, acetone, methyl alcohol, ethylene glycol and Phosphate buffer solution were purchased from Ke Long (Chengdu, China). Dracorhodin perchlorate , Sodium periodate and Ropivacaine hydrochloride was purchased from Shanghai McLean Biochemical Technology Co., Ltd. (Shanghai, China). Fetal bovine serum (FBS), DMEM (High Glucose) medium, 1% penicillin–streptomycin solution, and 3-(4,5-dimethylthiazol-2-yl)-2,5-diphenyltetrazolium bromide (MTT) assay were purchased from HyClone (Logan, UT, United States). All other reagents and solvents were of reagent grade.

### 2.2 Synthesis of quaternized chitosan

The QCS was prepared according to the method described by Xing (Gan et al., 2018) et al. CS (6 g) was completely dissolved in 200 ml of a 1% acetic acid solution at room temperature. Then 2–3 glycidyl trimethylammonium chloride (18.75 g) was dissolved in 50 ml of deionized water, followed by dropwise addition to the CS solution. The above mixed solution was placed in an oil bath at 80°C and stirred for 10 h. After the reaction was completed, the clear yellow solution was quickly poured into 100 ml of an ice-cold acetone solution, stirred well, and was placed in a 4°C refrigerator for 24 h. Subsequently, the remaining gel-like product was dissolved in 100 ml of methanol and precipitated in 125 ml of an anhydrous ethanol-acetone mixed solution (ethanol:acetone = 1:4). Finally, the precipitate was placed in a dialysis bag and dialyzed in deionized water for 1 week for purification, and the obtained completely clear solution was lyophilized using a freeze dryer to obtain purified QCS. The samples were stored in a −20°C refrigerator.

### 2.3 Synthesis of oxidized dextran

The OD was prepared using a classical polysaccharide-oxidation strategy (Guan et al., 2021). Briefly, 5 g of dextran was dissolved in 250 ml of acetate buffer solution at room temperature, and sodium periodate was added at a molar ratio of 1:1. The mixed solution was stirred and reacted at 42°C in the dark for 2 h. Then, ethylene glycol was added and stirred for 1 h to terminate the oxidation. Subsequently, it was purified by dialysis against deionized water for 3 days. The obtained transparent product was lyophilized by a lyophilizer and stored at −20°C.

### 2.4 Formation of polymerized dopamine @OD complex

Next, 0.1 g of dopamine (DA) was directly dissolved in an OD solution (1.5%, w/v) and stirred for 30 min. The DA was polymerized in solution to form PDA. The self-polymerized PDA interacted with the OD through the Schiff base and non-covalent bonds. A PDA@OD complex was formed.

### 2.5 Preparation of CS@BSA@DP drug-loaded nanoparticles (NPs)

This approach was slightly modified from a previous study by Han et al. (Han et al., 2015). First, the BSA-NPs were obtained using the dropwise desolvation method. Anhydrous ethanol (40 ml) was added dropwise to the BSA solution (10 mg/ml, 10 ml) at room temperature and stirred for 12 h to desolvate the BSA to obtain the BSA-NPs. Subsequently, 50 ml of a CS solution (1 mg/ml) was quickly poured into the BSA-NPs solution to obtain CS-coated BSA-NPs through electrostatic interaction, i.e., CS@BSA-NPs. The obtained CS@BSA-NPs were purified by washing three times with deionized water after centrifugation. To prepare the DP-loaded CS@BSA@DP-NPs, a certain amount of DP was dissolved in an anhydrous ethanol solution in advance. The DP-loaded CS@BSA@DP-NPs were prepared as described above.

### 2.6 Synthesis of integrated hydrogels

Next, 5 ml of the PDA@OD complex solution was obtained at room temperature. Similarly, the QCS was dissolved in deionized water to obtain 5 ml of the QCS solution (1.5%, w/v), and a certain amount of CS@BSA@DP-NPs was dispersed in the QCS solution. Then, the mixed solution of the QCS@NPs and PDA@OD complex solution (in a volume ratio of 1:1) were mixed, and after thorough mixing, the solution immediately changed from liquid to colloid.

### 2.7 Drug release from hydrogels

The integrated hydrogel containing the drug NPs was prepared as cylindrical samples with a diameter of 1 cm and height of 1 cm. Ropivacaine hydrochloride was substituted for the DP to measure the drug release. The samples were immersed in 10 ml of the medium and shaken gently at 100 rpm and 37°C. At certain time intervals on days 1, 2, 4, 8, 14, 20, and 26, a 3-ml measurement was taken, and the withdrawn volume was replenished with fresh medium. The medium was prepared using a 1 mg/ml lysozyme phosphate-buffered saline (PBS) solution with pH = 7.4 and pH = 6.8. To eliminate interference from the BSA, a UV-Vis spectrophotometer (UV-2250) was used to measure the concentration of ropivacaine hydrochloride at 263 nm. The cumulative release was calculated to determine the drug release profile and elucidate the drug release mechanism.

### 2.8 Characterization of physicochemical properties of hydrogels

The chemical group changes in the polysaccharides and hydrogels were investigated using a Tensor II Fourier transform infrared (FTIR, Bruker, Germany) spectrometer. The spectra were recorded in the wavenumber range of 4,000–500 cm^−1^. The proton-nuclear magnetic resonance (1H NMR) spectra of the samples were recorded on a Quantum i-400 NMR spectrometer (China) at 400 MHz to record the changes in the functional groups before and after polysaccharide modification. A multiscale 4e dynamic light scattering analyzer (Beckman Coulter United States) was used to measure the nanoparticle zeta potential and particle size. The microstructures of the hydrogels and NPs after freeze-drying were observed using a SU8010 field emission scanning electron microscope (JSM 6390). The hydrogels were freeze-dried prior to observation, and then a razor blade was used to cut the hydrogel sample to expose the cross-section and observe after spraying gold. The sprayed gold was used to improve the electrical conductivity and observe the morphologies of the NPs.

### 2.9 Rheological testing of hydrogels

Dynamic rheological tests were performed using a rheometer (MC2302; China). The prepared hydrogel (*D* = 20 mm, *H* = 3 mm) was placed on the probe head (*D* = 20 mm) of the rheometer and relaxed to a normal force of 0. Strain amplitude sweeps (0.01%–100%) were first performed to determine the stable linear viscoelasticity interval at 25°C. Then, a dynamic frequency sweep was performed at an angular velocity of 0.1–100 Hz with a strain amplitude of 1%.

### 2.10 Measurement of swelling ratio

The hydrogel was prepared in a cylindrical shape with a height of 1 cm and diameter of 1 cm, and the original mass Wd of the hydrogel was recorded using an analytical electronic balance (JA 2003, China). Hydrogel samples were placed in PBS solution (pH = 6.8) at 37°Cand completely immersed. The samples were removed at the set time points, and the mass Ws of the samples was recorded to observe the swelling changes of the hydrogel. Before weighing, the water on the surface of the hydrogel was wiped off using filter paper. Four parallel samples were in each group. The mass swelling ratio (SR) calculation formula was as follows:

SR=((Ws−Wd)/Wd)×100.

(calculate the swelling rate of the hydrogel contacting the liquid surface on one side by the same calculation method).

### 2.11 Performance test of *in vitro* degradation rate

The hydrogel was prepared in a cylindrical shape with a height of 1 cm and diameter of 1 cm. First, the dry weight of the freeze-dried hydrogel was weighed and recorded as Mo; then, hydrogels of the same shape and weight were placed in a lysozyme solution of pH = 7.4 (1.0 mg/ml) and lysozyme of pH = 6.8, respectively. In the enzyme solution (1.0 mg/ml), the hydrogel was removed at a predetermined time point, and the surface was wiped dry, freeze-dried, weighed, and recorded as Mi, with three parallel samples in each group. During this period, the degradation solution (prepared using PBS) was replaced regularly. The calculation formula for the hydrogel degradation rate was as follows: Degradation =((Mo-Mi)/Mo) × 100.

### 2.12 Characterization of adhesion properties

Fresh pig skin was used as a test substrate. In particular, 500 ul of the hydrogel was injected into the middle of a pair of the same base material (2.5 × 2.5 cm). Then, there was a 30-min wait for the combination to stably form a gel. The gel was tested with a universal chemical testing machine (Instron 5567, United States) with a loading force of 10 N and tensile rate of 10 N at 2 mm/min. The test ended when the hydrogel sample fell off of the base material on either side. The adhesion strength (As) was calculated as follows: As=F/A (where F is the maximum load of the adhesion and A is the surface area of the sample).

### 2.13 Characterization of antioxidant properties

First, the solvent was prepared as a methanol solution with a concentration of 0.04 mg/ml 2,2-diphenyl-1-picryl-hydrazyl-hydrate (DPPH) solution. Then, 3 ml of the DPPH solution was mixed with three hydrogel samples. Each sample had a diameter of 10 mm and thickness of 2 mm, was protected from light, and was placed in an oven at 37°C. The pure DPPH solution served as the control. The DPPH solution was aspirated after the expected time. Taking the methanol solution as a blank baseline, the absorbance of the DPPH solution was measured using a UV spectrophotometer at a wavelength of 516 nm. The calculation formula for DPPH free radical scavenging efficiency was as follows: DPPH scavenging (%) =((AB-AS)/AB × 100 (where AB represents the absorbance of the DPPH solution, and AS represents the absorbance of the DPPH solution after reacting with the sample).

### 2.14 Injectability of hydrogels

The QCS mixed with the CS@BSA@DP-NPs and PDA/OD solutions were loaded into different channels of a dual-channel syringe, and injected through a screw needle. If the hydrogel mixed solution was injected with a single-channel syringe premixed for approximately 5 s, a hydrogel prepolymer was added into the syringe channel and squeezed (needle diameter:29G).

### 2.15 Self-healing of hydrogels

Two identical cylindrical hydrogels were prepared, one of which was colored. The two pieces of hydrogel were cut into two pieces separately, and then the different-colored hydrogel sections were simply put together to ensure that the cross-sections completely adhered. The self-healing ability of the hydrogels was observed after 3 min.

### 2.16 *In vitro* biocompatibility test

#### 2.16.1 Fibroblast toxicity test

Four groups of samples were used in the experiment: QD, QPD, QPDN, and QPDN@DP hydrogel extracts. The hydrogel groups were named according to their final composition; for example, the QD hydrogel stands for QCS + OD, QPD stands for QCS + PDA@OD, QPDN stands for QCS + PDA@OD + CS@BSA/NPs, and QPDN@DP stands for QCS + PDA@OD + CS@BSA@DP/NPs. Each hydrogel extract was stabilized by the hydrogel and then soaked in a medium (volume ratio of hydrogel to medium: 1:10). The extract was filtered through a filter head and stored at 4 C. First, the cells with a cell density of 1.5 × 104 cells/well were blown into a 48-well plate. After 6 h, the cells were fully adherent and grown. The whole medium was aspirated from the experimental group and replaced with 1 ml of the hydrogel extract. The cell viability was detected by the of 3-[4,5-dimethylthiazole-2-yl]-2,5-diphenyltetrazolium bromide (MTT) method after 1, 3, and 5 days of co-culturing. After 1 and 3 days of co-culturing, calcein-AM/propidium iodide live-dead staining was performed to observe the cell morphology and proliferation by confocal laser microscopy (TCSSP5, Lecia, Germany).

The MTT detection method was as follows. After 1, 3, and 5 days of culturing, the medium in the well plate was removed and washed once with the sterile PBS solution. Then, 200 ul of an MTT solution (5 mg/ml) were added to each well. The wells were protected from light, and the well plate was placed into a 37°C cell incubator. After 4 h, the liquid inside was carefully removed to avoid removing the precipitate. Then, 200 ul of biological-grade dimethyl sulfoxide was added. The wells were protected from light, and the well plate was placed in a shaker at 37°C. After 30 min of reaction, 100 ul of the solution was aspirated into a 96-well plate, and the absorbance was detected using an enzyme-linked immunosorbent assay (WD-2102A, Liuyi Biotechnology, China) at a wavelength of 570 nm.

#### 2.16.2 Hemocompatibility test

Four groups of samples were used in the experiment: QD, QPD, QPDN, and QPDN@DP hydrogel extracts. Briefly, fresh rat whole blood containing a sodium citrate solution (3.8%) was centrifuged to obtain concentrated red blood cells (RBCs). The concentrated RBCs were diluted with normal saline to a 10% diluted RBC solution. Then, 500 ul of diluted RBCs were added to the hydrogel, with three parallel samples per group. In addition, 100 ul of 0.1% Triton-X and 100 ul of normal saline were used as the positive and negative controls, respectively. After incubation at 37°C for 3 h centrifugation at 2,000 rpm for 15 min, the supernatant was carefully aspirated and the absorbance was measured at 545 nm using a microplate reader. The hemolysis percentage was calculated as follows: Hemolysis (%) =((DS-DN)/(DP-DN)) × 100 (where DS is the OD value of the sample, DN is the OD value of the negative control, and DP is the OD value of the positive control).

### 2.17 Antibacterial test

Three groups of samples were used in the experiment: QD, QPD, and QPDN@DP hydrogel groups. The hydrogels were purified by alternate immersions in ultrapure water and absolute ethanol. Then, bacteria with a bacterial density of 10^8^ ((CFU)/ml) were blown into the hydrogel surface in the 48-well plate and co-cultured with the hydrogel. After culturing for 24 h, the bacterial suspension in 100 ul of the well plate was sucked into a 96-well plate, and the enzyme was used. The absorbance was measured at 570 nm by a standard instrument, and the bacterial mortality was calculated according to the OD value. In particular, 20 ul of the above-mentioned bacterial suspension was co-cultured for 24 h and spread evenly on the solid medium for 12 h. The bacterial survival rate on the plate was then calculated.

### 2.18 *In vivo* hemostasis experiment

All protocols were approved by the animal research committee of Southwest Jiaotong University (SWJTU-2208-SCS(070). Three groups of samples were used in the experiment: QD, QPD, and QPDN hydrogel groups. We used a hemorrhagic liver mouse (Sprague-Dawley (SD) rat, 200–300 g, female) to evaluate the hemostatic potential of the hydrogel (Hickman et al., 2018). Briefly, the mice were anesthetized and mounted on surgical soft plates. The liver of each mouse was exposed through an abdominal incision, and the surrounding serum was carefully handled. A piece of pre-weighted filter paper was placed on the parafilm below the liver. A scalpel was used to draw a bleeding opening with a depth of approximately 0.5 cm and length of approximately 1 cm. The skin plug plate was inclined at approximately 30°, and the hydrogel solution was immediately applied to the bleeding site using a syringe. After 1 min, the weight of the filter paper containing the absorbed blood was measured and compared with that of the control group (untreated after bleeding).

### 2.19 Preparation and experimental method of rat perianal abscess (infection) wound model

All protocols were approved by the animal research committee of Southwest Jiaotong University (SWJTU-2208-SCS(070). The specific methods of the wound and infection model preparation were as follows (Jiang et al., 2017). The SD rats were weighed and recorded before the experiment, and 3% sodium pentobarbital (0.3 ml/100 g rat body weight) was intraperitoneally injected for anesthesia. A small animal razor was used to shave the back of the rat as much as possible after anesthesia, and then a depilatory cream was used to thoroughly remove the hair. After 2 min, the rats were washed with normal saline and dried. The aforementioned steps were convenient for the follow-up wound to be fixed with a film. After the depilation treatment was completed, the backs of the rats were fixed on a small animal test bench. During the experiment, the body temperature of the rats was maintained at (36.5 ± 0.3)°C, and the back of the rat was disinfected with 10% povidone-iodine. To prepare for surgery, the hole diameter of the skin punch was adjusted to 1.5 cm, and the punch and skin contact head were sterilized. Then, the skin was punched on both sides of the back spine of the rat (one hole on one side, while ensuring that the infection foci did not affect each other). During the process, sterility was ensured, and the drilling depth reached the destruction of the muscle layer. The wound site was wiped with sterile gauze or a cotton swab to perform the initial hemostasis treatment on the wound. 100 μL of the bacterial solution (*Escherichia coli* 10^8^ colony forming units (CFU)/ml) was added to the wound. An indwelling needle film was used to properly seal the wound to ensure that the film could be closely attached to the skin, and a clinical sterile tape was used to fix the film around the film to ensure that the bacterial fluid was in contact with the wound tissue for a sufficient time. The dressing was removed 48 h after exposure to the infection. Pus and secretions were visible to the naked eye, and the skin at the wound edge was slightly swollen. After the formation of the infection foci, normal saline was used for preliminary debridement. The wounds of the experimental group were cleaned and disinfected, and 200 µL of the corresponding hydrogels were injected into the wounds. After disinfection, 200 ul of sterile saline was injected. The wounds were sprayed with *Escherichia coli* 100 ul/10^6^(CFU)/ml at a fixed time every day, and then were rinsed with normal saline to simulate fecal contamination during daily defecation. All experimental rats were injected with antibacterial drugs postoperatively to reduce the risk of infection. The ,morphological observations and pathological analysis were conducted as follows. In each group, the surrounding tissue was surgically removed on the 7th and 14th days after operation, respectively. When retrieved, the animals were sacrificed at specific time points and the wound healing state was photographed. For the histopathological studies, both the hydrogels and tissue samples were formalin-fixed, embedded in paraffin, and stained with hematoxylin and eosin (H&E) and Masson’s trichrome. The inflammatory responses and collagen fibril expressions were examined and assessed by a blinded pathologist, using a combination of well-established pathological staining assessment protocols.

### 2.20 Statistical analysis

The Statistical analysis was performed using the GraphPad Prism software (version 8.0). The differences between groups were determined using a one-way analysis of variance with Tukey’s test. The differences were considered statistically significant at *p* < 0.05, and *, **, and *** were considered as *p* < 0.05, *p* < 0.01, and *p* < 0.001, respectively, in the quantitative images. The results are expressed as the mean ± standard deviation.

## 3 Results and discussion

### 3.1 Preparation and structural characterization of hydrogels

**FIGURE 1:**
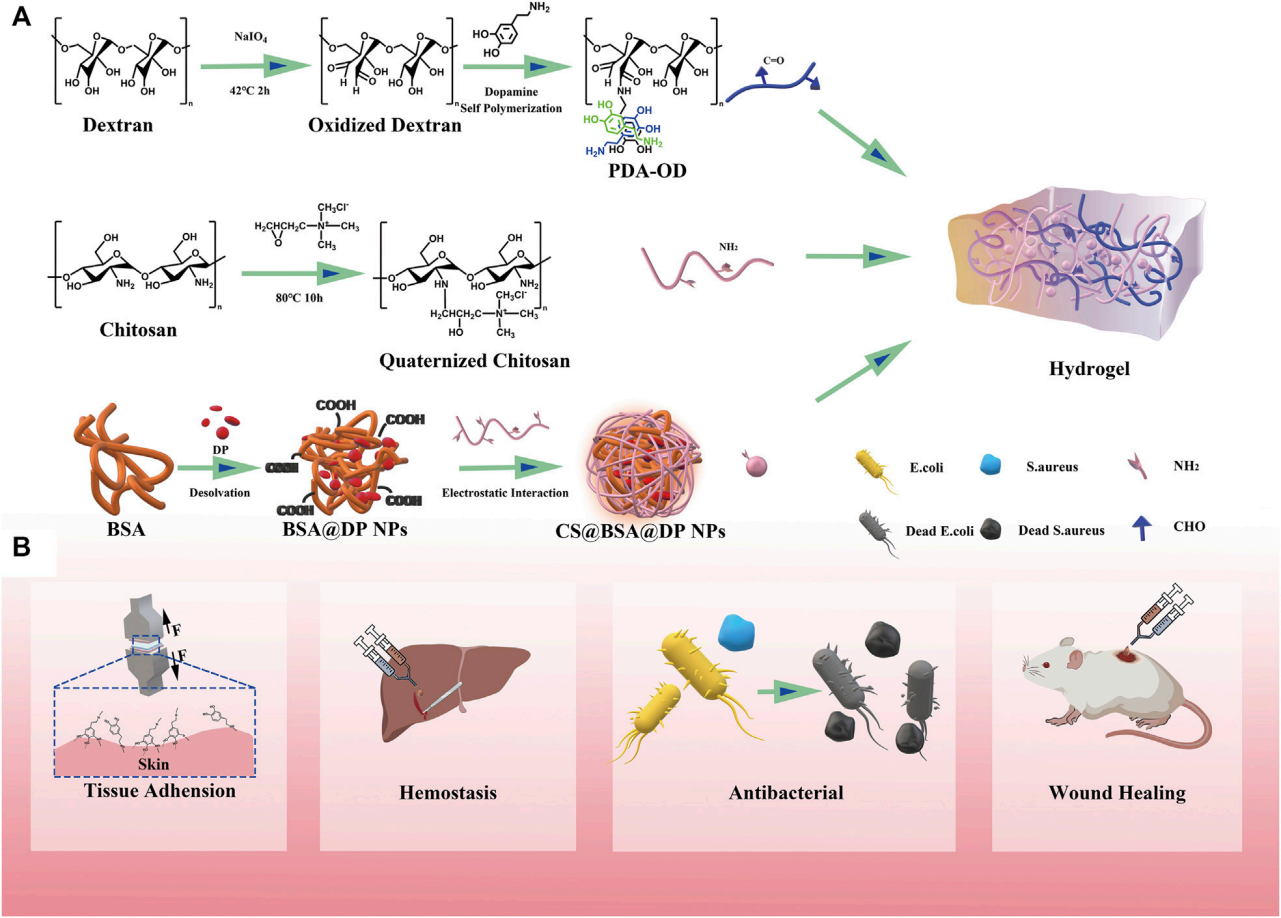
Illustration of a multifunctional all-in-one adhesive hydrogel for treating perianal infectious wounds. **(A)** Synthesis scheme of QPDN@DP hydrogel. **(B)** Schematic illustration of the main functions of the hydrogel.

This article describes antibacterial, adhesive, hemostatic, and healing hydrogels for the treatment of perianal infectious wounds. The hydrogels in this study are based on a simple Schiff base reaction and catechol groups between PDA, QCS, and OD, and the CS@BSA@DP/NPs are cross-linked by Schiff bases and hydrogen bonds. Briefly, the CS@BSA@DP/NPs are directly dispersed in the QCS solution, and the mixture is thoroughly mixed with a homogeneous mixture of PDA-premixed OD to form QPDN@DP hydrogels. Compared with other complex-formed hydrogels, the entire process of hydrogel formation in this study is fast and simple, and does not require the involvement of toxic chemical cross-linking agents. We hope to prepare a multifunctional “all-in-one” drug-loaded hydrogel dressing with multiple functions, such as rapid self-healing, injectable free-formability, superior wet tissue adhesion, antibacterial activity, hemostasis, and loading of healing-promoting drugs to guide regenerated infected wounds and restore normal skin anatomy and physiological functions (Figure 1).

**FIGURE 2:**
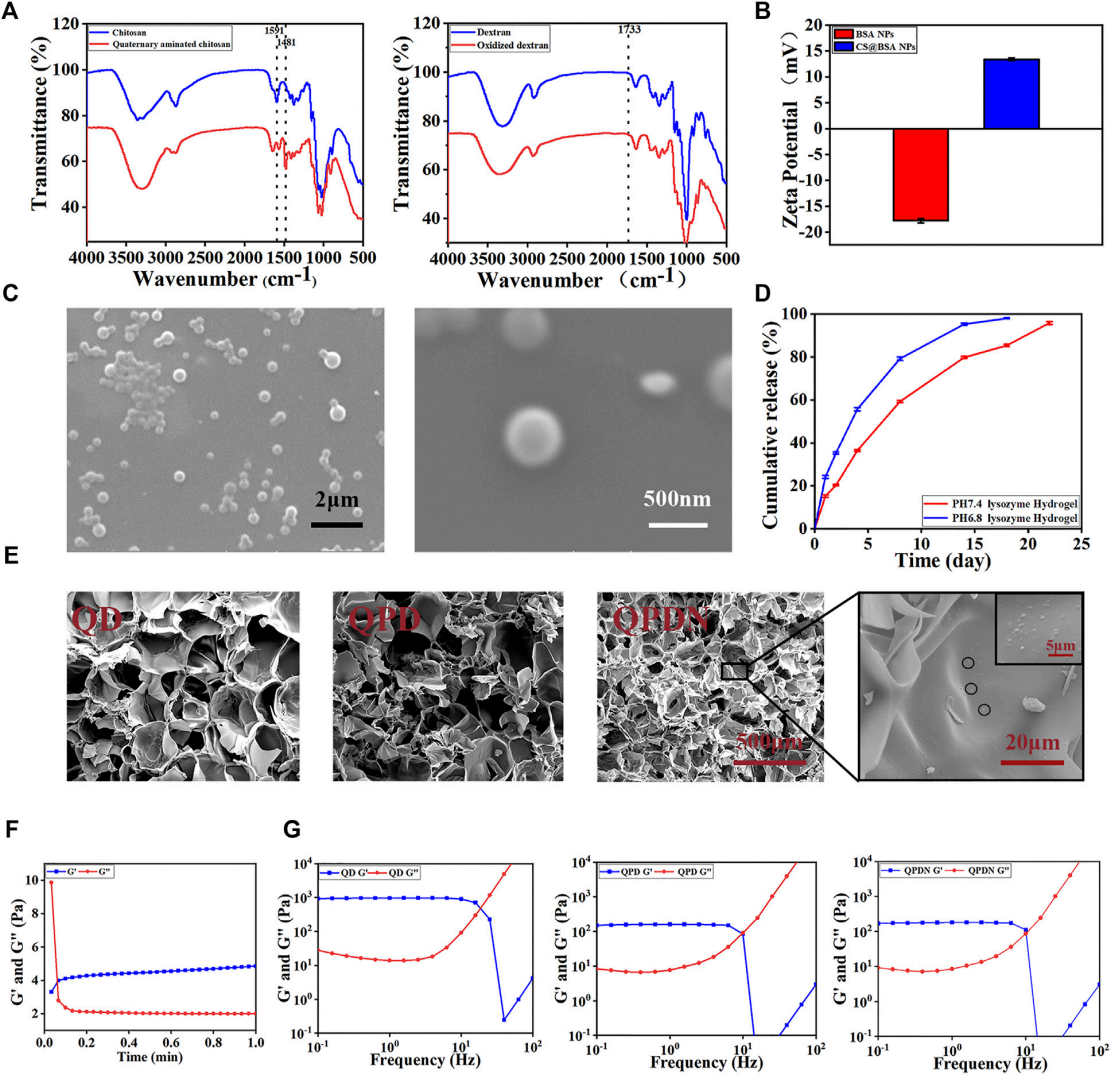
Preparation and physical characterization of hydrogels.**(A)**FTIR spectra of QCS and OD.**(B)**Zeta potential of the BSA-NPs and CS@BSA-NPs. **(C)**SEM image of CS@BSA-NPs, scale bar:2 μm; 500 nm. **(D)**Drug release from hydrogels in lysozyme solutions at pH = 7.4 and pH = 6.8. **(E)** SEM shows the microstructure of the hydrogel cross-section along with the nanoparticle. Scale bar:500,20, 5 μm. **(F)** Gelation time of the QPDN as determined by rheological analysis at 37°C. **(G)** Oscillatory shear rheology of the hydrogels.

First, the chemical structure of the material was investigated using FTIR and 1H NMR spectroscopy. The results are shown in Figure 2A. Natural CS (blue curve) shows a peak at 1,591 cm^−1^, i.e., located at the bend of the N-H bond of the original amino group. After modification (red curve), the peak disappears. It is observed at 1,486 cm^−1^ as a new sharp peak corresponding to the methyl group of the quaternary ammonium salt, consistent with the reports in the literature. In the H-NMR spectrum of QCS (S1), the absorption peak of H on the three methyl groups in the branched group of the introduced quaternary salt appears at 3.23261. The experimental results show that the quaternization modification of the CS is successful (Zhao et al., 2015).

For OD, in which the broad peak at 3,800–3,000 cm^−1^ represents the stretching vibration of -OH, the peak intensity of OD decreases compared to that of dextran, while a new peak appears at 1733 cm^−1^ The absorption peak corresponds to the hemiacetal structure. This is because the aldehyde group formed by the hydroxyl oxidation of the dextran interacts with the hydroxyl group on the molecular structure of the dextran, thereby forming a hemiacetal structure. This also confirms that an oxidation system is obtained (Wang et al., 2021).

The CS@BSA@DP/NPs loaded with the hydrophobic Chinese medicine molecules were prepared using the desolvation method. Scanning electron microscopy (SEM) micrographs show that the CS-coated BSA-NPs are spherical (Figure 2C). The average particle size of the CS@BSA-NPs is 342.5 nm, as determined by dynamic light scattering. The polydispersity index is 0.059, indicating that CS@BSA-NPs have a uniform particle size distribution (S2). The zeta potential of the BSA-NPs uncoated with CS is −17.8 mV. After coating with CS, the zeta potential changes from negative to positive (+13.4 mV), proving that the CS was successfully coated onto the BSA-NPs (Figure 2B).

The CS coating on the BSA-NPs serves two purposes. First, the CS coating stabilizes the BSA-NPs. Uncoated BSA-NPs are unstable for a long time in aqueous media and easily decompose, Glutaraldehyde (GA) is often used for crosslinking and stabilization; however, GA is toxic (Han et al., 2015). In this study, CS with good biocompatibility was used to encapsulate the GA through the electrostatic effect of rapid mixing, so that it had good biocompatibility. Second, the CS coating contains amino groups that can react with the aldehyde groups in the OD to uniformly cross-link in the hydrogel network, while increasing the hydrogen bonds to strengthen the cross-linking of the hydrogel.

The drug release results show that the integrated hydrogel achieves a uniform release of ropivacaine hydrochloride (Figure 2D). First, the drug in the NPs is slowly released from the degradation of the CS@BSA-NPs. Second, the released drugs diffuse into the 3D network structure of the hydrogel. The covalent Schiff of the hydrogel slows the degradation of the base and ensures it is gradually released into the medium, which further controls the burst release of the drug.

**FIGURE 3:**
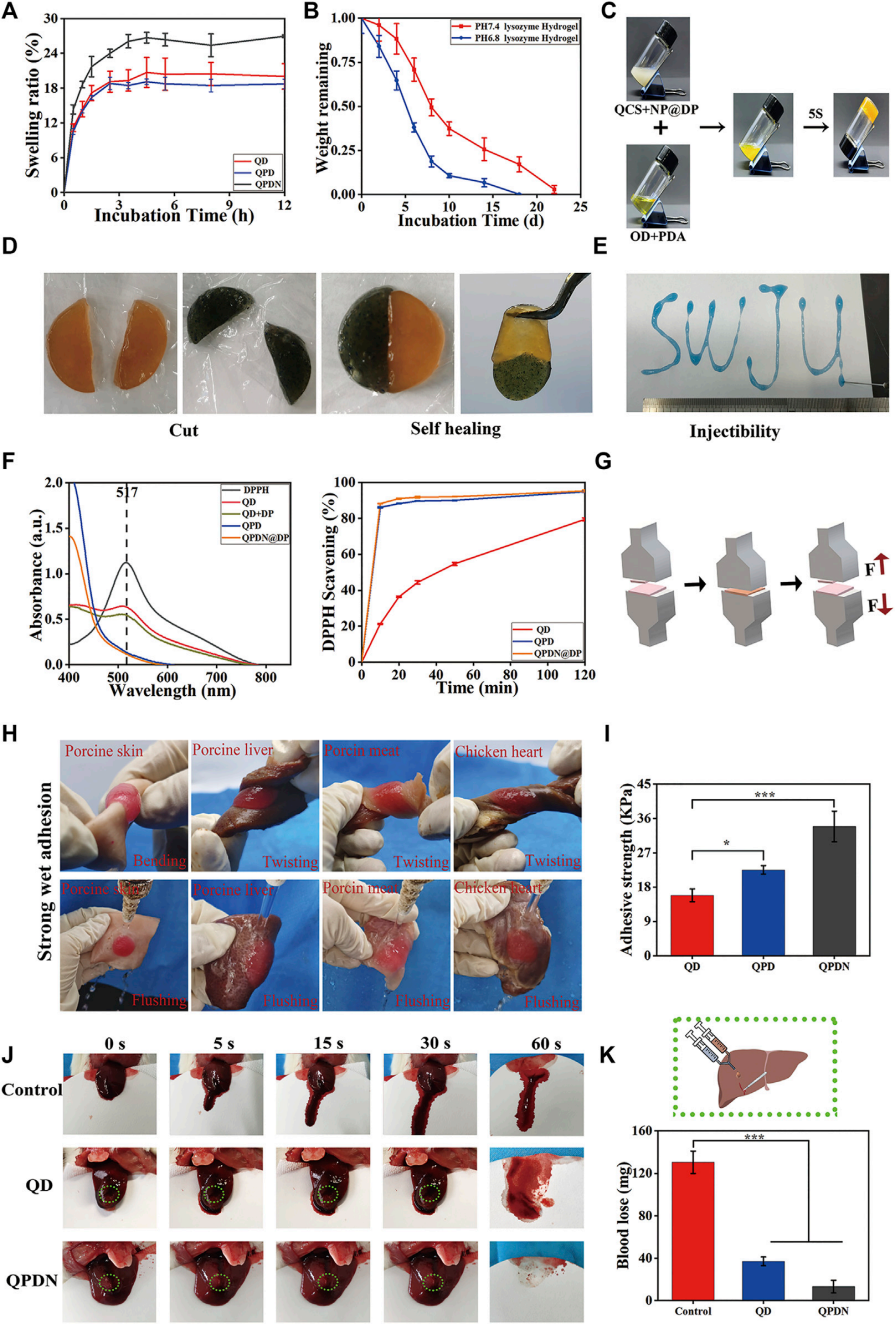
**(A)**Swelling properties. **(B)**Degradation profiles. **(C)**The optical images of hydrogels *via* a tube inverted test. **(D)**Appearance of self-healing process between two different colored semicircle hydrogels. **(E)**The injection process of QPDN hydrogel, SWJU was drawn. **(F)**Clearance curve of DPPH at 30 min and The radical scavenging rate of hydrogel in DPPH solution. **(G)**Schematic diagram of adhesion measurement. **(H)**Photographs of wet adhesion of QPDN hydrogels on different tissues. **(I)**Comparison of Adhesion Strength of Hydrogels in Each Group **(J)**Photographs of liver wound with different treatments at predetermined time intervals (0, 5, 15, 30, and 60 s) **(K)** Schematic diagram of liver hemorrhage treatment; Total blood loss from rat liver wound after 60 s with different treatments. Mean ± SD (*n* = 3). *p < 0.05, **p < 0.01, ***p < 0.001.

We estimated the gelation time via tube inversion experiments (Figure 3C). As shown, the gel speed increases with increasing concentrations of QCS and OD (S4). As the viscosity of the solution increased with an increasing QCS concentration, we chose 3% (w/v) as the gelling concentration. The addition of an appropriate amount of PDA accelerated the gelation time, confirming the participation of the PDA and promoting hydrogel formation. Therefore, we chose a 1% (w/v) DP premix. An NPs input amount of 0.2% (w/v) loaded with drug content was considered as the best choice for promoting tissue proliferation (Jiang et al., 2017).

The microstructure of the hydrogel did not change after rapid lyophilization. The SEM images in Figure 2E show that all of the hydrogels exhibit a three-dimensional network structure. The 3D porous network structure of the hydrogel facilitates the absorption of interstitial fluid and blood, and is beneficial for preventing pus from spreading and hemostasis. The QD hydrogel has a large pore size (200 μm) and smooth surface. In the presence of the PDA, the QPD hydrogel has a well-interconnected microporous three-dimensional structure with microfibrils at the edges of the pores, indicating that the PDA chains are well connected to QCS and OD. The microfibrils in the QPD hydrogels, which are the typical morphologies of PDA-based hydrogels reported in previous studies (He et al., 2020), form a complex network that mimics the structural diversity of the extracellular matrix. However, the addition of the NPs enhances the crosslinking of the hydrogel, resulting in a reduction in pore size (100 μm). This highly porous hydrogel can lead to a uniform drug release during slow diffusion. Furthermore, the porous interconnected structure provides sufficient space for cell growth, attachment, proliferation, and secretion from the extracellular matrix (Hoque et al., 2018).

### 3.2 Gel behavior and rheological properties of hydrogels

The hydrogel time is critical in biomedical applications for hemostasis. To further investigate the gelation time and post-gelling stability of the QPDN@DP hydrogels, we preloaded the QCS@NPs and PDA@OD solutions in a double-barreled syringe and used a flow-through syringe after the solutions were extruded from the syringe. The gel time was recorded using a rheometer (Figure 2F). Notably, the double-barrel syringe had a needle with a mixing channel to ensure adequate mixing of the QCS/NPs and PDA@OD solutions. The storage modulus G′ and loss modulus G″ at 37°C were recorded with the rheometer. When G″ was greater than G′, it indicated a liquid property, whereas when G’ was greater than G”, it indicated a colloidal property. It was observed that under the recording of the rheometer, the mixed solution changed from a liquid state to a gel state in only approximately 5 s. In addition, the storage modulus and loss modulus remained in a stable state after gelation, indicating that the hydrogel is stable in gelation. This gelation time is also suitable for even coverage of the gel on perianal deep, narrow, and long wounds.

To further confirm the stability of the hydrogels, we performed rheological tests on the QD, QPD, and QPDN hydrogels at 25°C (Figure 2G). A rheometer was used to detect the storage modulus (G′) and loss modulus (G″) of the hydrogels at a fixed amplitude γ (1%) and different angular frequency ω values to obtain the rheological properties of the hydrogels. The rheological results show that each group of hydrogels exhibits typical viscoelastic behavior, because each group of hydrogels show stable G′ and G″ values when the frequency is increased from 0.1 to 10 Hz, and the storage modulus (G′) is larger than the loss modulus at all frequencies (0.1–10 Hz). The addition of the PDA makes the inherently brittle structure of the hydrogel network soft and elastic, as it introduces recoverable noncovalent interactions in the hydrogel network. These can dissipate energy during the process. The mechanical properties of the hydrogel are further enhanced by the addition of the NPs. Therefore, the final hydrogel exhibits good mechanical properties and softness, similar to natural soft tissue, to satisfy the requirements for skin defect repair.

### 3.3 Swellability and biodegradability

Owing to the hypertonic environment of infected wounds, the water absorption capacity of the hydrogel is a key parameter in choosing a suitable dressing. However, an excessive swelling rate compresses the wound, causing both compressive pain in the wound and tissue ischemia, and is not conducive to wound repair. Therefore, proper swelling control is extremely important. The swelling ratios of the QDs, QPDs, and QPDNs were measured over time (Figure 3A). As shown, during the first 1 h, the water absorption of all three hydrogels increases. After 3 h, the growth curve gradually becomes smooth. The swelling curves show that the water absorption of the QPDN hydrogels increased slightly with the addition of the NPs, and the final swelling ratios of QD, QPD, and QPDN are 18.72%, 20.04%, and 26.96%, respectively. The results show that the designed hydrogel has a suitable water absorption capacity, i.e., it can absorb wound exudates (such as blood and inflammatory factors) and maintain a moist microenvironment without affecting healing owing to excessive swelling and compression of the wound surface. This provides an ideal protective barrier for wound repair. For the situation of unilateral contact wounds, the physical and measurement results are in Supplementary Material S1, the results are consistent.

An ideal hydrogel should be degraded after hemostasis and wound healing. The *in vitro* degradation behavior of the hydrogels was investigated by monitoring their weight loss in PBS containing lysozyme at different pH values (Figure 3B). We found that all hydrogels experience a relatively rapid initial degradation during the first week, and then their weights gradually decreased over time. The pure natural polymer hydrogel is completely degraded. It could be completely degraded in approximately 22 days in the degradation solution of pH = 7.4, but it was degraded in 18 days in the degradation solution of pH = 6.8. This shows that our hydrogel is completely degraded. In addition, it has a pH-responsive release. The fully degraded hydrogel can directly alleviate the pain caused by secondary injury from the patient’s dressing during the skin repair process.

### 3.4 Injectability and self-healing properties of hydrogels

Similar to most Schiff base-bonded hydrogels, the macroscopic injectability and self-healing properties of the QPDN hydrogels were confirmed. More specifically, the dyed QPDN master mix can be continuously injected into A4 paper using a 1-ml syringe, and a formed “SWJU” font can maintain a hydrogel state, as shown in Figure 3E. This unique injectability is ideal for deep and narrow wounds. Finally, the two cut pieces of the QPDN hydrogel were fused within 30 min without any external intervention, and the healed hydrogel maintained its integrity even after external force was applied to the hydrogel (Figure 3D). Again, we tested the stability of the self-healing hydrogel with a rheometer, measuring the storage modulus (G′) and loss mode of the hydrogel at a fixed amplitude γ (1%) and different values of the angular frequency ω (G″). The results show stable G′ and G″ values that almost coincide with those of the intact hydrogel (S5). Taken together, these results demonstrate the excellent injectability and self-healing ability of the QPDN hydrogels to facilitate simple, rapid, and effective coverage for irregular and deep wounds.

### 3.5 Hydrogel scavenging oxygen free radicals

Free radicals play a vital role in all the stages of wound healing. The topical application of free radical-scavenging materials has been shown to accelerate wound repair (Crunkhorn, 2020). The DPPH scavenging efficiency was used to estimate the antioxidant activity of the hydrogels. From Figure 3F, we can see that the QD hydrogel slowly scavenged approximately 78% of the free radicals within 120 min, whereas the hydrogel with PDA added scavenged approximately 86.43% of the free radicals in approximately 10 min. The 10-min biological clearance rate of the hydrogels with DP NPs reached 88.25%. The QPDN@DP hydrogel eliminated almost all of the free radicals in approximately 30 min, reflecting the synergistic antioxidant effect of the TCM molecules and hydrogel system. In conclusion, the antioxidant activity of the QPDN@DP hydrogel makes it a contender for skin wound repair dressings.

### 3.6 Tissue adhesion and *in vivo* hemostatic ability of hydrogels

Good tissue adhesion plays a very important role in the wound healing process, especially in perianal infected wounds. As the anus is the main outlet of excrement, it is frequently exposed to bacteria and other pollutants. Clinically, the wound surface is often cleaned after defecation, causing the drug dressing to fall off and resulting in repeated dressing changes. Surprisingly, the QD, QPD, and QPDN hydrogels showed excellent skin adhesion. Pigskin was used to test the biotissue adhesion ability of the hydrogels using a universal chemistry testing machine (Figure 3G). The adhesion strength of QD is approximately 15.86 kPa, the adhesion strength of QPD is approximately 22.54 kPa, and the adhesion strength of QPDN is approximately 33.91 kPa. The addition of the PDA enhances the adhesion ability of the hydrogel, and may be related to the presence of a large amount of DP. The phenolic functional group not only participates in the formation of various non-covalent bonding forces (such as hydrogen bonding, π-π stacking, and cation-π interactions), but can also undergo disproportionation reactions to generate coupling to increase the adhesion strength. After the addition of the NPs, the adhesion strength of QPDN exceeds that of QPD and the adhesion ability is further increased, owing to the increase in viscosity from the further strengthening of hydrogen bonding and polyphenol group interactions by the NPs (Teng et al., 2021). To further characterize the wet tissue adhesion of QPDN, we prepared fresh biological tissues such as pig skin, pig liver, pork, and chicken heart, injected the dyed hydrogel premix on the surfaces of the above biological tissues, and stabilized the gel for approximately 30 s. After bending, twisting, and other operations on the tissue adhering to the hydrogel and scouring it in water, we observed that the hydrogel was intact and did not detach from the surface of the biological tissue with the series of violent operations (Figure 3H). This shows that our hydrogel dressing can firmly adhere to the wound surface and cannot easily fall off; thus, it can exert its effect for a long time. This is consistent with the care needs of perianal infection wounds, that is, to clean up the contaminants on the surface of the hydrogel, but not to cause the separation of the hydrogel from the tissue. In addition, it avoids the need to change the hydrogel dressing frequently, and allows the drug-loaded hydrogel to work on the wound surface for a long time. Thus, this adhesive and antifouling hydrogel dressing is ideal.

The adhesion of hydrogels is crucial for hemostasis in biomedical applications (Li et al., 2021). Of the four stages of wound repair, hemostasis is the first (which also shows its importance in the overall repair process). Hydrogels with adhesive properties can seal wounds immediately and exhibit good hemostatic properties (Saini et al., 2016). We evaluated the hemostatic effect of these hydrogels using the SD rat liver bleeding model, and selected the QD and QPDN@DP hydrogels as representatives. As shown in Figure 3J, in the untreated group, nearly 130 mg of blood is shed from the liver of SD rats; the QD hydrogel group has one-third of the blood loss (37 mg) of the control group, and the QPDN hydrogel shows less bleeding in the glue group (13.3 mg) (Figure 3K). With enhanced adhesion, the QPDN group hydrogel shows an excellent hemostatic effect in the liver hemorrhage model, almost closes the hemorrhage port at the time of liver hemorrhage, and firmly adheres to the hemorrhage surface. In addition, the oxidized catechol group of the DP in QPDN can increase the interaction between the hydrogel and tissue and adhesion to blood cells and platelets by forming covalent bonds with nucleophilic groups on the tissue surface (Wang et al., 2021). There is a significant difference in bleeding volume between the control and QPDN hydrogel groups (*p* < 0.001), demonstrating the excellent *in vivo* hemostatic ability of the QPDN@DP hydrogel.

### 3.7 *In vitro* antibacterial

**FIGURE 4:**
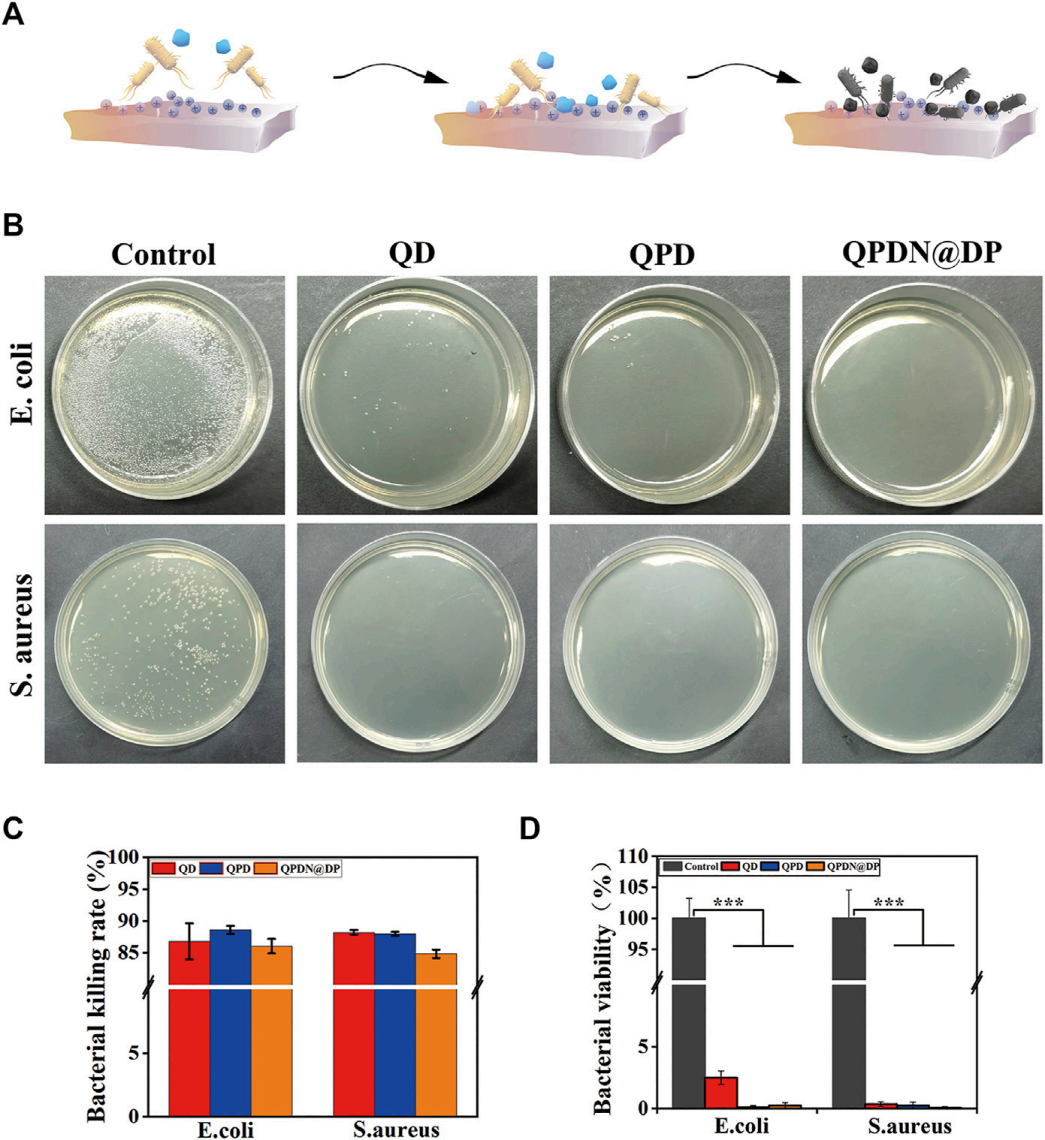
Antibacterial activity of the hydrogels. **(A)** Schematic diagram of the principle of contact antibacterial. **(B)** Antibacterial performance of the presented treatments against S. aureus and E. coli as determined by CFU counts. **(C)** Hydrogels sterilization rate. **(D)** Bacterial viability. Mean ± SD (*n* = 3). *p < 0.05, **p < 0.01, ***p < 0.001.

We investigated the antibacterial properties of the QD, QPD, and QPDN@DP hydrogels. Unlike the previously reported antibacterial hydrogels, our hydrogels exhibited excellent contact antibacterial activity. The QCS in the backbone has potent and broad antibacterial activity against a range of gram-negative and gram-positive bacteria (Figure 4). The effective antimicrobial properties suggest that antimicrobials are critical for repairing skin and promoting wound healing, especially in preventing bacterial inflammation.

### 3.8 Biocompatibility

Biocompatibility is crucial for hydrogels in biomedical applications (Gan et al., 2018). First, the toxicity of the hydrogel was assessed.

**FIGURE 5:**
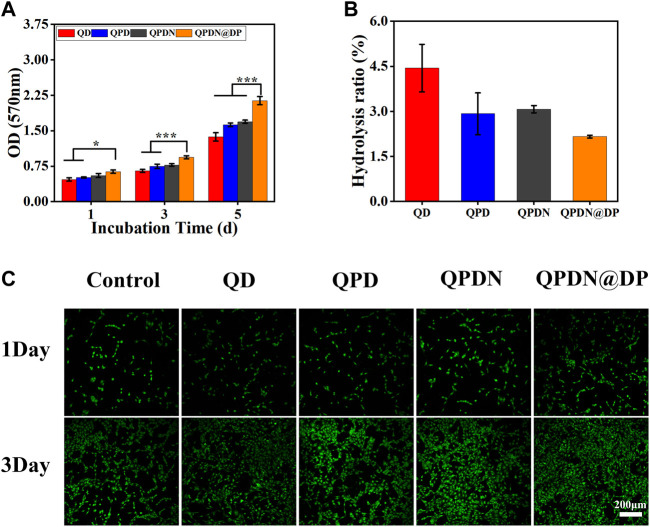
*In vitro* biocompatibility of hydrogels. **(A)** L929 cell viabilities after 1, 3, and 5 days incubation with leaching solution of hydrogels. **(B)**Hemocompatibility of hydrogels. **(C)** Live/dead staining results of L929 cells cultured for 1–3 days after different leaching solution of hydrogels (scale bar = 50 μm). Mean ± SD (*n* = 3). *p < 0.05, **p < 0.01, ***p < 0.001.

By MTT analysis (Figure 5A), based on the increase in the number of L929 cells after incubation with leachate from each group of hydrogels for 1, 3, and 5 days. The results indicate that the hydrogels have good cytocompatibility, and the number of cells in the QPDN@DP group has the highest proliferation effect compared to the other groups. Live/dead cell staining was used to validate the results of the MTT assay. As shown in Figure 5C, most of the L929 cells in the test group are viable, and few dead cells are observed. Simultaneously, the proliferation status is different among the experimental groups. Cells proliferate owing to the adsorption of PDA; in the QPDN@DP group, the cells evidently proliferate and spread the best, and the cell state is superior. Subsequently, we investigated the hemocompatibility of this material, as it would come into contact with blood at the wound site when used as a dressing (Figure 5B). The hemolysis rate of all hydrogel samples was <5%, i.e., a safe level for hemostatic materials (Saini et al., 2017). These results indicate that each hydrogel has excellent biocompatibility and can be used as a wound dressing in tissue engineering.

### 3.9 Infectious wound healing experiment

In contrast to acute wounds, which heal over time, wounds that come into contact with infected skin heal slowly (over 8 weeks or more) or do not heal at all (Liang et al., 2019). The first step in wound management involves debulking the wound to remove necrotic and/or infected tissue. During the later stages, for the postoperative treatment of perianal infectious disease, it is important to avoid frequent and complex bacterial contamination and biofilm formation to limit infection while reducing persistent inflammation in chronic skin wounds to control scarring tissue hyperplasia, thereby avoiding the occurrence of a series of malignant complications such as anal stenosis. The skin can regenerate in wounds at epidermal and partial thickness levels through its self-healing function (Kaplani et al., 2018). However, in infected full-thickness skin wounds, the self-healing function of the skin cannot function normally because of the destruction of a large number of regenerative elements in the skin epithelium (Tang et al., 2021). To verify the effect of QPDN@DP on the healing of infectious full-thickness skin wounds *in vivo*, we established a rat model of a continuous infectious skin injury, and during the whole experimental process, a bacterial solution with the concentration of *Escherichia coli* in feces was sprayed daily to simulate daily defecation exposure to bacteria. We observed the whole process for 14 days.

After 48 h of bacterial infection, yellow pus was observed on the wound surfaces of all model rats. H&E staining also showed that the lower muscle layer of the defect site was blurred, and the surrounding edema was evident. Various signs indicated that the infection model was successfully established (S8). After the modeling was successful, the hydrogel was injected for treatment.

**FIGURE 6:**
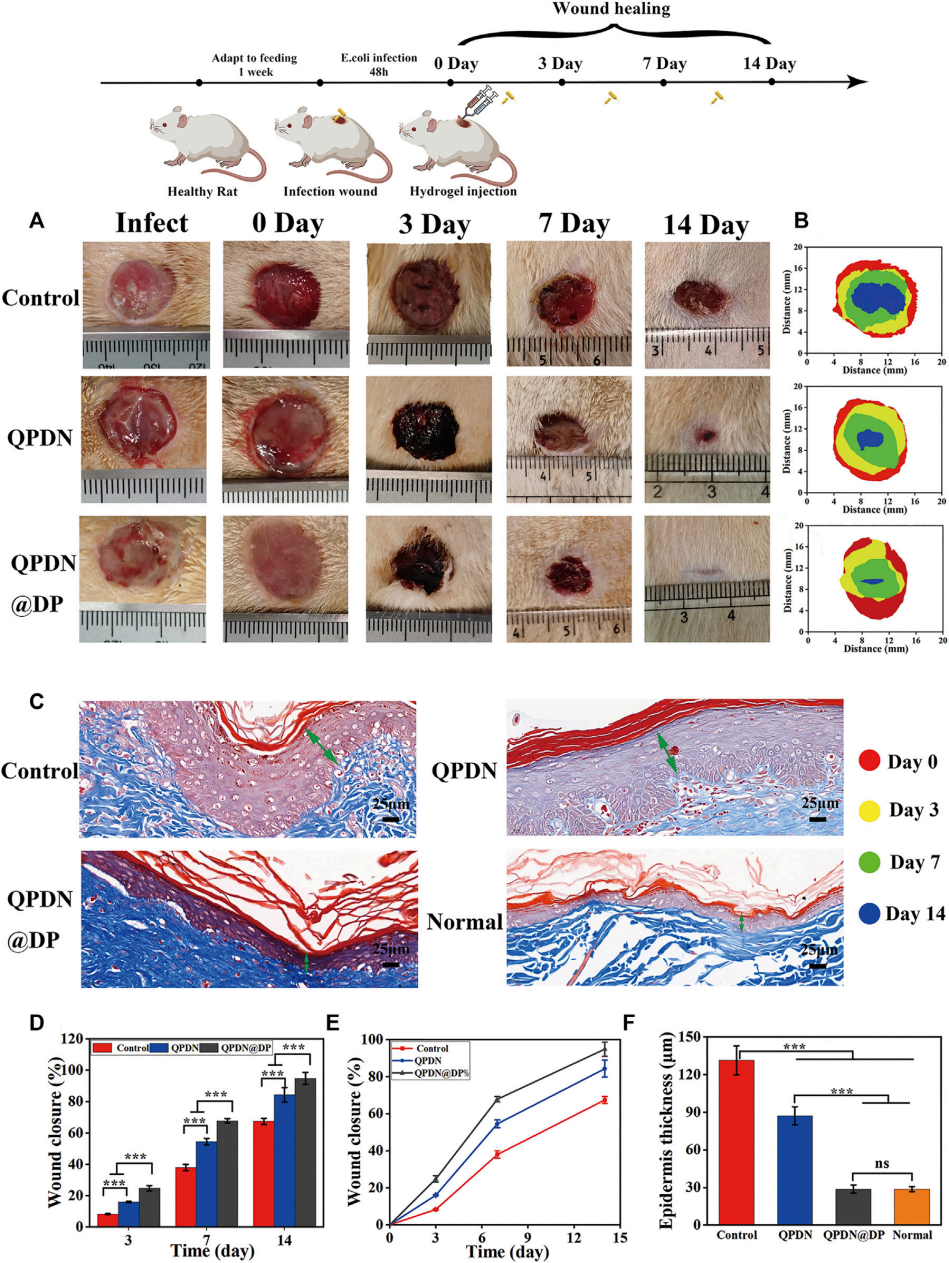
Repair of animal model of wound after perianal infection.**(A)** Schematic illustration for constructing continuous infected wound and healing process; Representative photographs of the continuous infected wound healing process of rats under different treatments (*n* = 3). **(B)** Wound fractions healed by different treatments on days 0, 3, 7, and 14 (*n* = 3). **(C)**Masson staining of wounds on day 14, green arrows represent epidermal thickness (Scale bar = 25 μm) **(D,E)** Quantitative analysis of continuous infected wound area in each group (*n* = 3). **(F)** Quantitative analysis of epidermal thickness in each group on day 14 in Masson staining (*n* = 3). Mean ± SD (*n* = 3). *p < 0.05, **p < 0.01, ***p < 0.001.

Both the QPDN and QPDN@DP groups were able to form a gel at the wound site within 5 s and adhered tightly to the skin, sealing the defect well and achieving wound hemostasis. We also found that the wound defects in the QPDN and QPDN@DP groups decrease faster than those in the blank control group, and the wound closure is faster than that in the blank group. As shown in Figure 6A,B, photographs of the wound healing status of the rats were taken on different days, and the wound size was measured and quantified. We can see in the animal pictures of wound closure that the skin defects in the QPDN and QPDN@DP groups are basically closed at approximately 14 days. The QPDN@DP group has the best wound closure, whereas the blank group still has approximately 30% of the skin defects on the 14th day (Figure 6D,E). The area is not closed and remains in a defect state. On the 14th day of Masson’s trichrome staining (Figure 6C), the regenerated epithelium is partially thickened in the control group, and the QPDN group is also slightly thickened relative to the normal group, whereas the thickness of the regenerated epidermis in the QPDN@DP group is almost equivalent to that of normal rat skin (Figure 6F). This indicates that the QPDN@DP group hydrogel has a significant effect on preventing excessive hyperplasia after surgery and can prevent the formation of postoperative scars, thereby further preventing the occurrence of anal stenosis. These results further indicate that the QPDN@DP hydrogel has the best effect on promoting the healing of infectious wounds.

**FIGURE 7:**
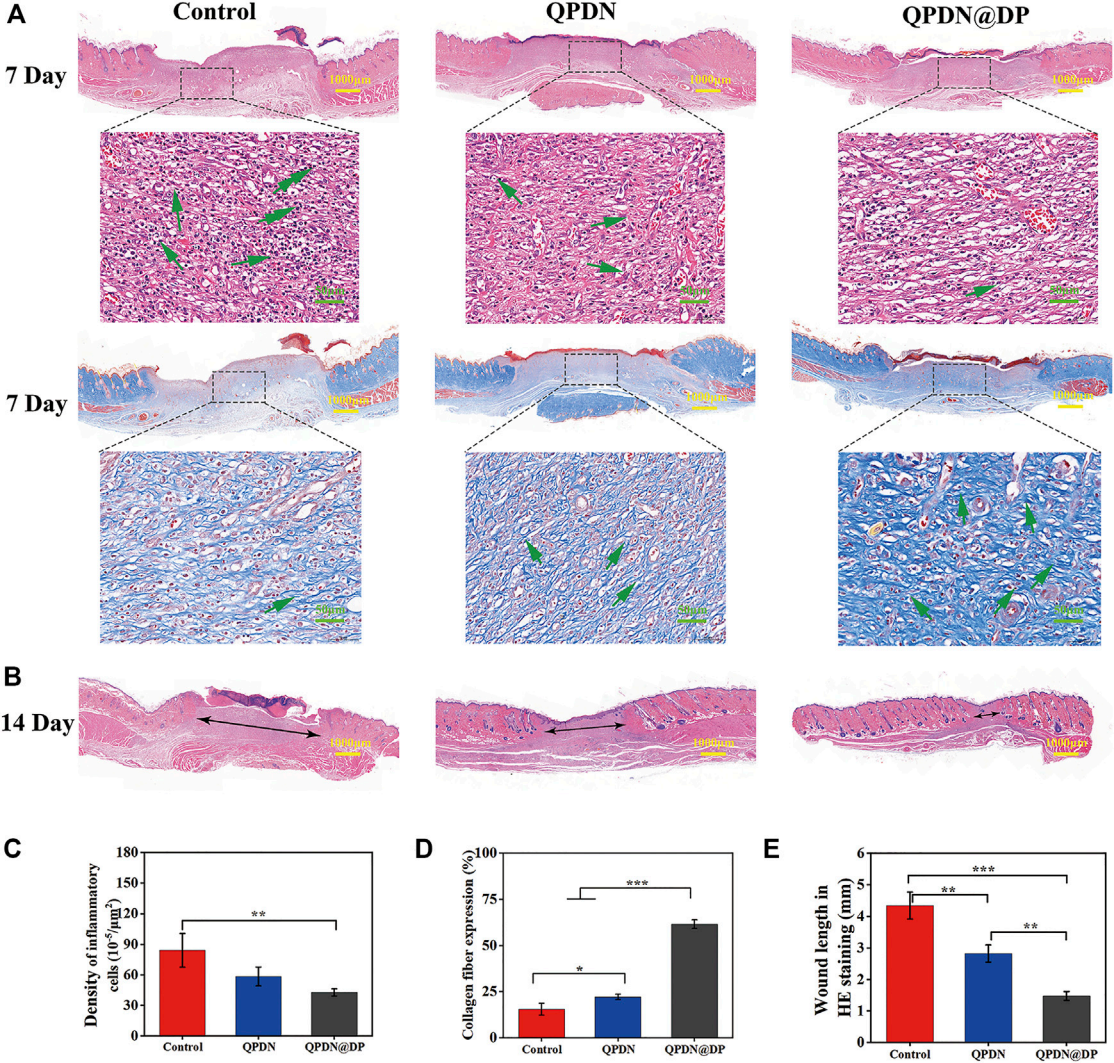
**(A)**HE and Masson staining of skin defects on day 7; In HE staining, Green arrows represent the inflammatory cells. In Masson staining, Green arrows represent the collagen fibers (Scale bar = 100 μm; 50 μm) **(B)**HE staining of skin defects on day 14; Black arrows represent unhealed wound diameter (Scale bar = 1000 μm) **(C)**Quantitative analysis of inflammatory cells for different groups of HE staining on day 7. **(D)**Quantitative analysis the number of collagen fibers for different groups of Masson staining on day 7. **(E)**Quantitative analysis the diameter of wound for different groups of HE staining on day 14. Mean ± SD (*n* = 3). *p < 0.05, **p < 0.01, ***p < 0.001.

We performed a series of histopathological analyses on wound tissue sections using H&E and Masson’s trichrome staining to further verify that the accelerated wound healing *in vivo* was owing to the anti-inflammatory effect of the hydrogel, and to further evaluate the wound healing (Figure 7A). We analyzed the tissue sections of rats for 7 days. The enlarged images of the H&E-stained tissue sections under the microscope show that each experimental group has different degrees of inflammation. Under a 400x light microscope, we quantitatively analyzed the inflammatory cell density in each group, which revealed the excellent anti-inflammatory properties of the QPDN@DP hydrogel (Figure 7C). In addition, because collagen fibers are an important part of wound repair, Masson’s staining was used to determine the percentage of collagen fiber expression area to evaluate the treatment effect. The control group reached 15.89%, the QPDN group 22.15%, and the QPDN@DP group 61.53%. The blue fibrous structure gradually deepens in the image, indicating an increased collagen fibril content (Figure 7D). Compared to the control group, the collagen fibers in the QPDN@DP group are arranged in a dense and orderly manner; therefore, the significant difference indicates that the QPDN@DP hydrogel could significantly accelerate the deposition of collagen. Finally, we measured the diameter of the incompletely healed wound diameter in 14-days HE staining (Figure 7B,E). Compared to the blank group, the QPDN@DP group shows a significant difference, and the degree of healing is the best.

The above experiments and analyses demonstrate that QPDN@DP hydrogels have excellent anti-inflammatory and tissue regeneration effects and exhibit excellent performance in promoting the healing of infected wounds *in vivo*.

## 4 Conclusion

In summary, we designed a novel and efficient synthetic strategy and successfully prepared composites with suitable swelling rates, rapid gel formation, fully degradable, self-healing properties, morphological plasticity, super-strong wet tissue adhesion, antifouling, and inherent antibacterial properties to promote healing. The QPDN@DP hydrogels show good hemocompatibility and cytocompatibility in hemolysis, live/dead cell staining, and MTT assays. In addition, the QPDN@DP hydrogel can adapt well to regular-or irregularly shaped wounds, and has sufficient hemostatic ability to treat skin wounds. Notably, the wound healing speed of the QPDN@DP group is faster than that of the blank and QPDN groups, indicating that the QPDN@DP hydrogel can be combined with insoluble Chinese medicine molecules. Chinese medicinal molecules can be released uniformly in this system, so that the two can be more scientifically synergistic. In summary, the QPDN@DP hydrogel dressing was prepared using a practical and simple method, providing a new method for the treatment of difficult-to-heal perianal infected wounds.

## Data Availability

The original contributions presented in the study are included in the article/Supplementary Material, further inquiries can be directed to the corresponding authors.
