# Supplementary material for: Multifunctional all-in-one adhesive hydrogel for the treatment of perianal infectious wounds
Source: Front Bioeng Biotechnol. 2022 Sep 30;10:989180. doi: 10.3389/fbioe.2022.989180 (PMC9561363; doi:10.3389/fbioe.2022.989180)
Supplement: Supplementary file 1 [file DataSheet1.doc]

**Support information**

**
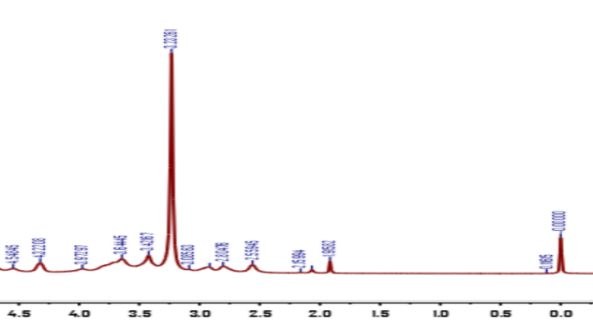
**

**Fig. S1.** ^1^H NMR of spectrum of QCS.

1. **The quaternization degree of QCS**

**The quaternization degree of QCS was measured according to the previous report. K2CrO4 (2.0 g) was dissolved in 5ml deionized water, dropped in a AgNO3 solution (0.1 mol/L) until the red deposit formed, left to rest for 12 h, and then the filtered solution was diluted to 100 mL. QCS (0.1 g) was dissolved in deionized water (50 mL) at stirring to form QCS solution. And K2CrO4 (1 mL) solution was added into QCS solution. Then, AgNO3 (0.1 mol/L) solution was dropped into QCS solution until the red deposit formed. The blank titration was used with other deionized water (50mL) to perform at the same condition. The formula of quaternization degree was calculated according the following equation:**

**DS(%)=(((V_2_-V_1_)×M×314))/(m_3_×10^3^)×100**

**where, DS is the substitution degree of QCS, V_1_ and V_2_ are the volume (mL) of AgNO3 standard solution that deionized water and QCS solution used, respectively. 314 is the molar mass (g/mol) of QCS. m is the weight (g) of QCS. M is the molar concentration (mol/L) of AgNO3 standard solution. The calculated degree of quaternization was 35.00.**

**2.Determination of oxidation degree of oxidized dextran (OD)**

**The hydroxylamine hydrochloride(8.69g) was dissolved in 75ml deionized water to form 0.25mol/l hydroxylamine hydrochloride aqueous solution, 0.2g oxidized dextran was dissolved in 25ml prepared hydroxylamine hydrochloride aqueous solution ,left to rest for 3 hours with 3 drops of methyl orange solution (0.1%) was added , then 0.1mol/l NaOH solution was dropped until the solution changes from red to bright yellow. Blank titration was carried out under the same conditions. The formula of aldehyde degree is as follows: Ad (%) = (((v_1_-v_0_) × M × 160))/(m_3_ × 10^3^) × Where DS is the degree of substitution of OD, and V_1_ and V_0_ are the volume of 0.1mol/l NaOH (ml). 160 is the molar mass of glucan (g/mol). M is the weight of OD (g). M is the molar concentration of NaOH standard solution (mol/l). The calculated oxidation degree of dextran is 95.00.**


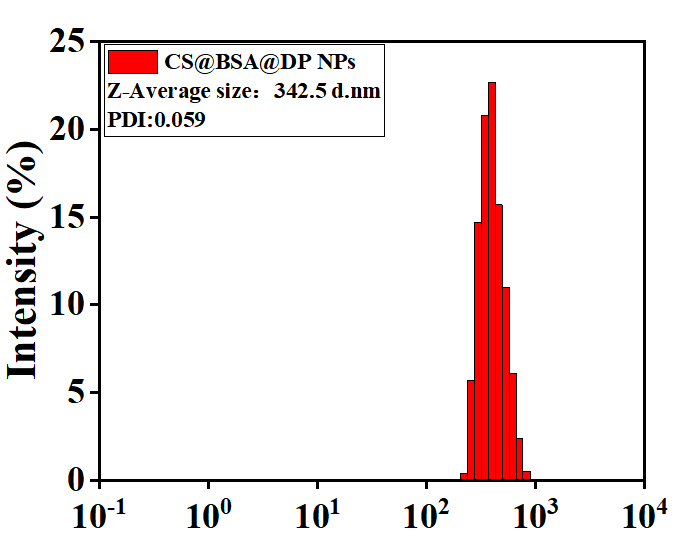


**Fig. S2.** Particle size of CS@BSA- NPs .


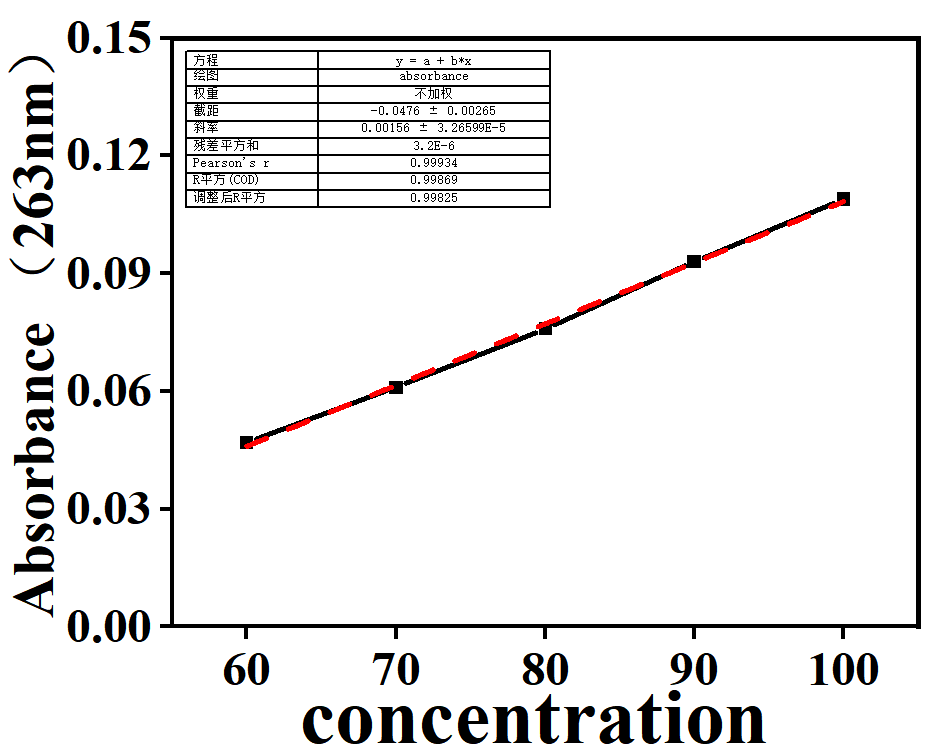


**Fig. S3.** Standard curve of ropivacaine hydrochloride


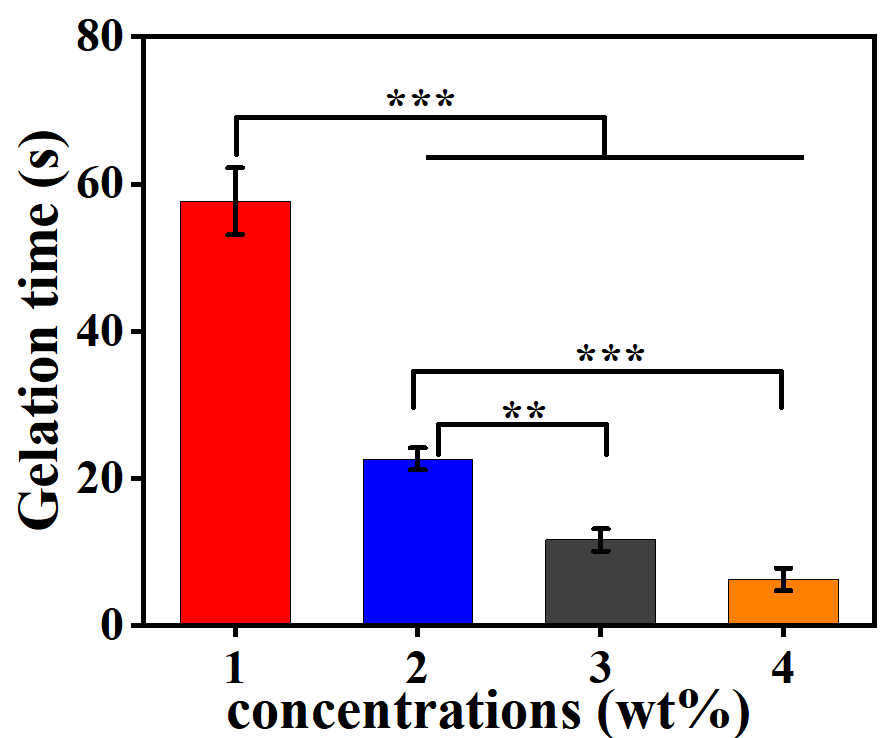


**Fig. S4.** Gelation time of different concentrations.


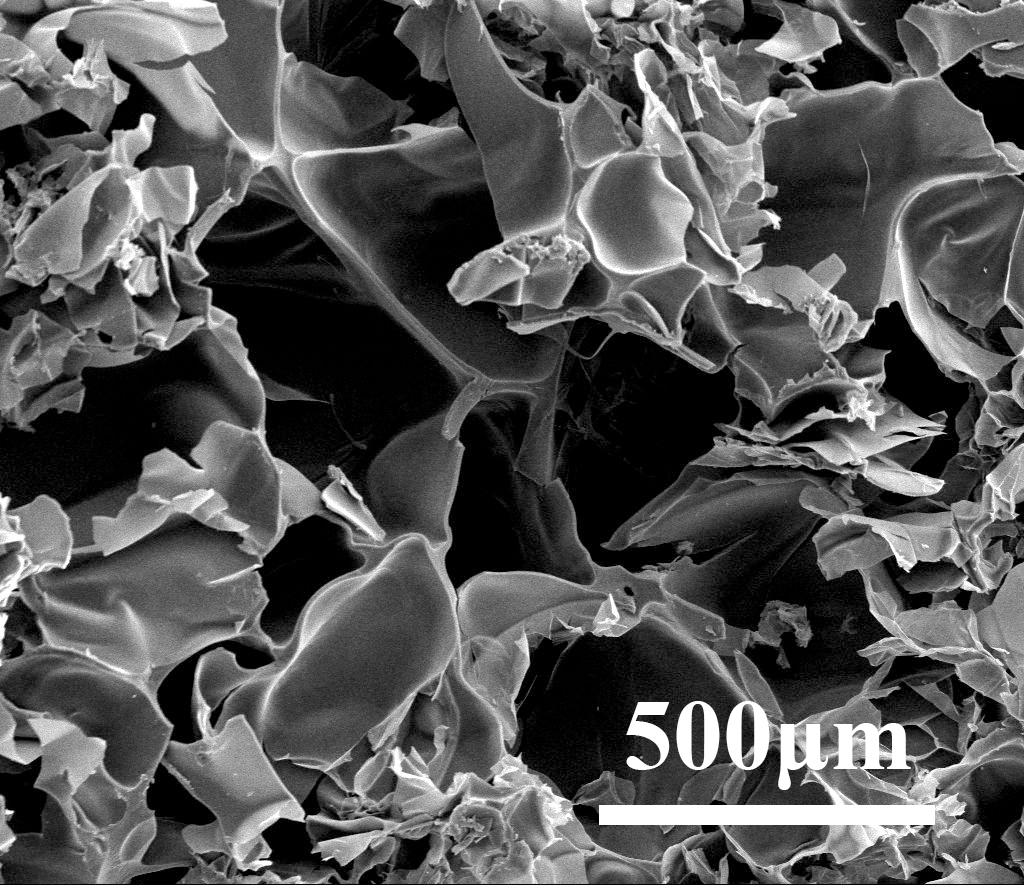

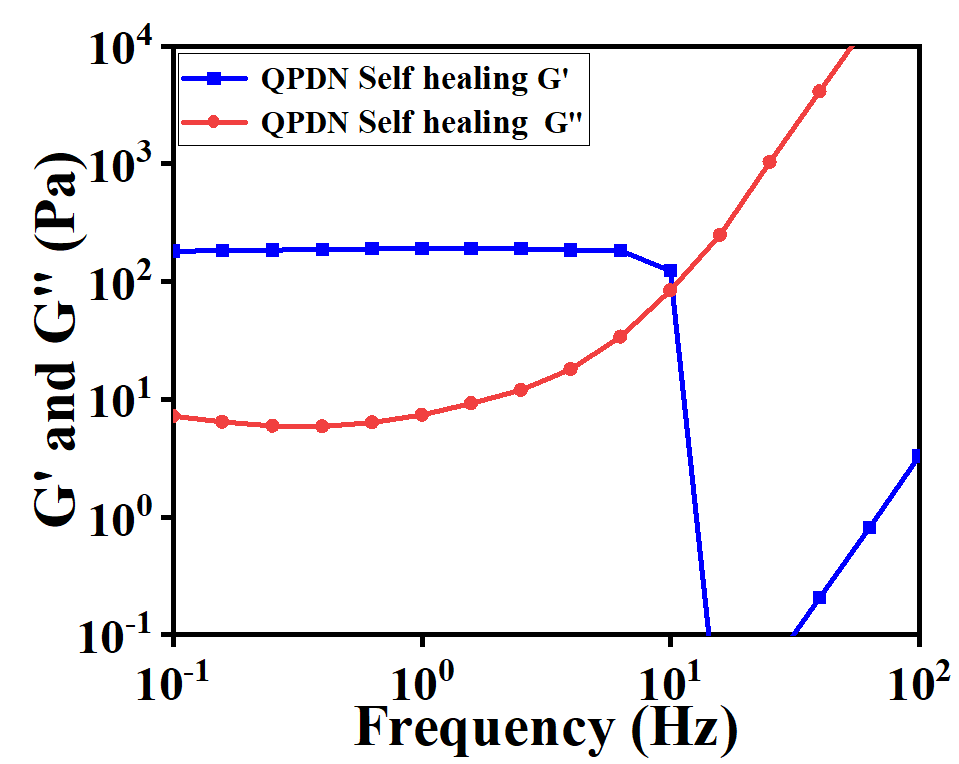


**Fig. S5.** Rheological properties and SEM of Self healing hydrogels


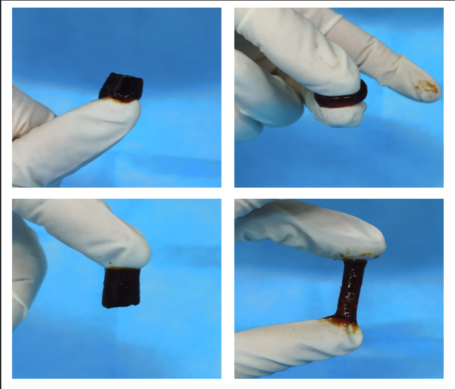


**Fig. S6.** Physical drawing of hydrogel compression and stretching


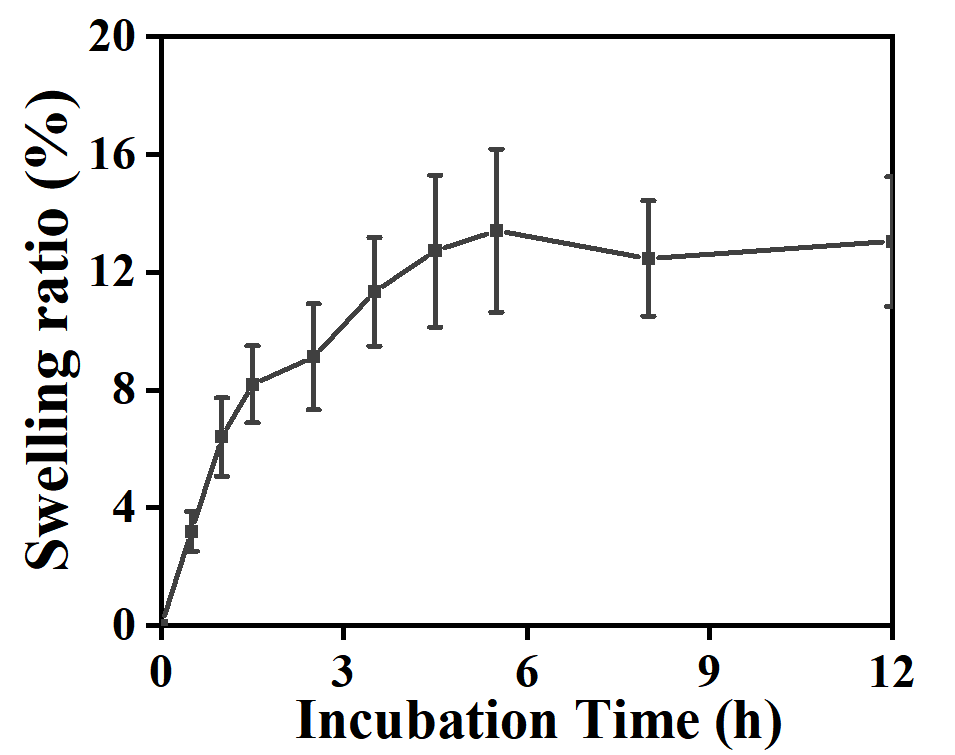

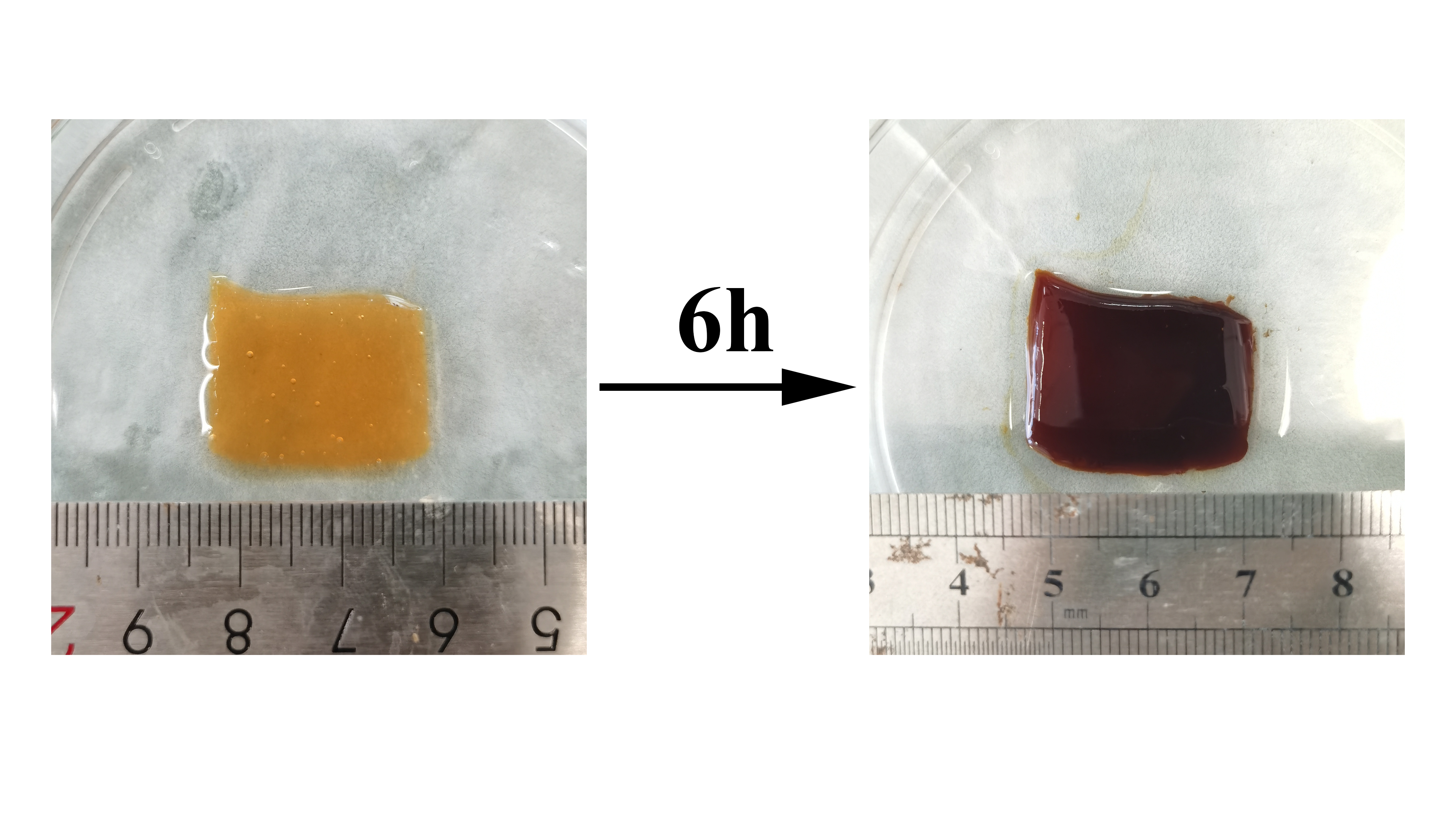


**Fig. S7.** Swelling ratio and physical map of hydrogel contacting liquid level on single side


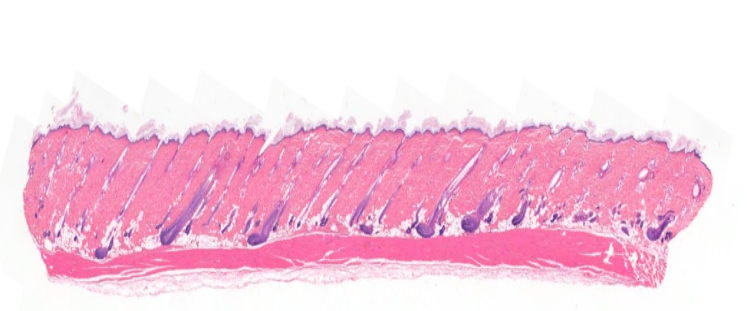


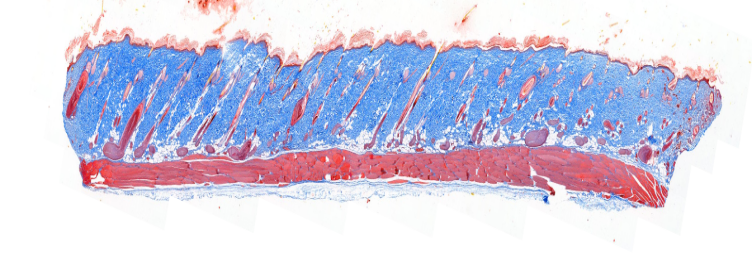


**Fig. S8.** HE staining and Masson staining of normal rat skin


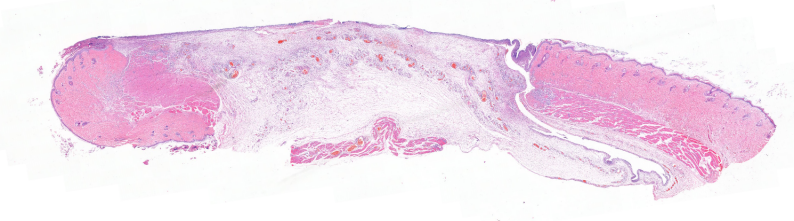


**Fig. S9.** HE staining infection rat skin
